# Efflux pump-associated antimicrobial resistance genes in *Staphylococcus* spp. from dairy and meat samples

**DOI:** 10.55730/1300-0152.2783

**Published:** 2025-10-06

**Authors:** Meryem Burcu KÜLAHCI, Sumru ÇITAK

**Affiliations:** Department of Biology, Faculty of Science, Gazi University, Ankara, Turkiye

**Keywords:** *Staphylococcus* spp., efflux pump, antimicrobial resistance, biocide tolerance, reserpine

## Abstract

**Background/aim:**

This study aimed to investigate the phenotypic resistance and distribution of efflux pump-associated antimicrobial resistance genes in *Staphylococcus* spp. isolated from dairy and meat samples. Antimicrobial resistance in foodborne bacteria increases with antibiotic exposure and biocides, particularly through efflux mechanisms. Thus, monitoring potential genetic reservoirs in the food chain is very important.

**Materials and methods:**

A total of 132 dairy and meat samples were collected for the study, and *Staphylococcus* spp. were isolated using Mannitol salt phenol red agar. Antimicrobial susceptibility was evaluated using the Clinical and Laboratory Standards Institute’s microdilution method. Twenty-six resistant isolates were identified by 16S rDNA sequencing. The effect of reserpine on MIC values was evaluated using microdilution tests to assess the role of efflux pumps in antibiotic resistance and biocide tolerance. Antibiotic resistance and efflux pump genes were detected using real-time PCR with specific primers.

**Results:**

Of the 77 isolates evaluated, 26 (33.8%) were resistant to at least one antibiotic. Resistance to tetracycline (69.2%) and cefuroxime (38.5%) were the most common. The administration of reserpine reduced minimum inhibitory concentration (MIC) values across all cefuroxime-resistant isolates and in a subset of tetracycline- and nitrofurantoin-resistant strains, suggesting the potential involvement of efflux pumps. It also lowered MICs for triclosan (46.7%) and povidone-iodine (32%). The most frequently detected efflux pump genes were *smr* (88.5%), *efrA* (84.6%), *efrB* (80.8%), *mdeA* (84.6%), and *norE* (80.8%). *qacA/B* was not detected in any isolate.

**Conclusion:**

Genes encoding efflux pump proteins were commonly found in *Staphylococcus* spp. isolated from dairy and meat samples. Reserpine inhibition tests confirmed the phenotypic effects of these genes. These results suggest efflux-mediated resistance can significantly impact antibiotic tolerance and biocides in foodborne isolates. Continued surveillance and control strategies are essential to limit the spread of these resistance genes in the food chain.

## Graphical abstract

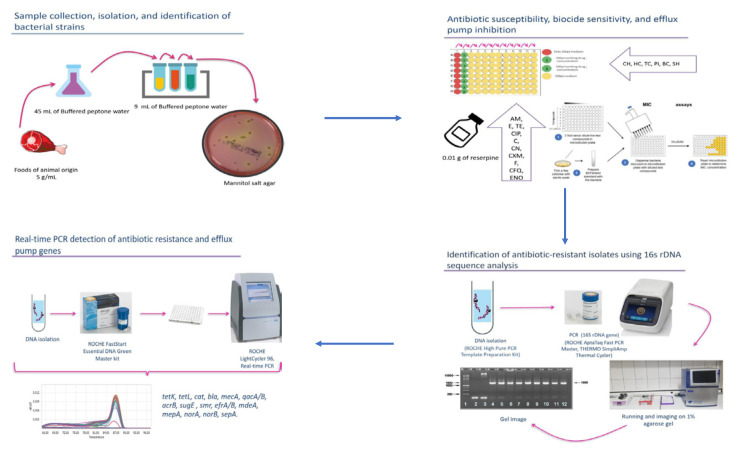

## 1. Introduction

The development of antimicrobial resistance in bacteria is a global challenge. It is largely driven by the inappropriate use of antibiotics for treatment or preventive purposes in animal infections and their use as productivity enhancers in animal nutrition. This means that antibiotics enter the food chain, and the use of related antibiotics in human medicine trigger defense mechanisms in bacteria, ultimately leading to the development of antibiotic resistance (Lange and Brokking, 2005; Yılmaz et al., 2016; Abbas et al., 2024). Studies have shown that colonization of pathogenic or normal bacteria carrying these resistance genes to the animal and human microbiome through food is an increasing threat to animal and human health (Salam et al., 2023).

Staphylococci are among the most commonly isolated contaminant bacteria from various food samples. *Staphylococcus aureus* causes various diseases, especially mastitis in animals and food poisoning in humans (Yurdakul et al., 2013). Although coagulase-negative staphylococci are considered commensal and contaminant bacteria, they are known to have pathogenic effects in humans (Rodrigues et al., 2017).

Cleaning and disinfection are of great importance to prevent microorganism contamination of food and the resulting foodborne infections (Pakdel et al., 2023). Biocides are used during raw food production. They are used to sterilize food production facilities and equipment, thereby inactivating bacteria that cause food spoilage (Marquez et al., 2017). There are concerns about whether the widespread use of biocides, and other antimicrobial agents, is driving the development resistance against them (Coombs et al., 2023). Bacterial resistance to biocides was detected in the 1950s and 1960s. This was when biocides started to be used for bacterial sanitation in the food industry. Although studies on biocide resistance are not as numerous as those on antibiotic resistance, it has become an issue of great importance in recent years (Lavilla Lerma et al., 2013).

Biocides used in the food industry are disinfectants containing chlorine, cationic quaternary ammonium compounds, alcohol-based disinfectants, biguanides, iodinated compounds, hexachlorophene, and triclosans (Kampf, 2018). However, the widespread and unconscious use of biocides in this sector leads to the acquisition of resistance genes by microorganisms or the adaptation of initially sensitive microorganisms, as with antibiotics. In addition, there is a growing danger of cross-resistance between antibiotics and biocides (Larsson and Flach, 2022).

One of the mechanisms known to cause antimicrobial resistance in bacteria is the overexpression of efflux pump protein genes. Efflux pumps play an important role in antimicrobial resistance. Recently, a growing number of multidrug and drug-specific efflux pumps have been characterized in different kinds of bacteria. There are 5 families of efflux pump proteins: the ATP-binding cassette (ABC) family, the multidrug and toxic compound extrusion (MATE) family, the major facilitator superfamily (MFS), the resistance-nodulation-division (RND) family, and the small multidrug resistance (SMR) family (Huang et al., 2022).

To prevent efflux pump-based resistance and to increase pump sensitivity, efflux pump inhibitors are an interesting class of compounds in the fight against resistance. Reserpine is an antihypertensive herbal efflux pump inhibitor known to inhibit P-glycoprotein (Sun et al., 2014; Aygül, 2015). Studies show that antibiotic resistance can be prevented by using efflux pump inhibitors (Fernandes-Fuentes et al., 2014; Sezgin, 2025).

In aim of the present study was to determine the status of antibiotic and biocide tolerance in *Staphylococcus* spp., in addition to evaluating the presence of reserpine-sensitive efflux pump and genes associated with antimicrobial resistance and efflux pumps. We focused on isolates obtained from dairy and meat samples collected from diverse markets within Ankara, Türkiye.

## 2. Materials and methods

### 2.1. Sample collection, isolation, and identification of bacterial strains

We purchased 132 dairy and meat samples, including 60 cheese samples, 13 raw milk samples, 18 chicken samples, and 41 minced meat samples, from different markets in Ankara, Türkiye. Approximately 5 g or 5 mL of each sample were mixed with 45 mL of sterile buffered peptone water (BPW, Merck, Darmstadt, Germany, catalog number: 107228) and homogenized. The bacterial suspensions were serially diluted in sterile BPW solution and spread on Mannitol salt phenol-red agar (Merck, catalog number: 105404) plates (Turkish Food Codex Microbiological Criteria Regulation, 2011)^1^. The plates were incubated aerobically at 37 °C for 48 h. From each plate, typical pink and yellow staphylococci colonies were isolated and cultured. The preliminary identification was carried out using basic biochemical tests. Isolates were stored in 20% glycerol at −80 °C (Chajecka-Wierzchowska et al., 2015).

### 2.2. Antibiotic susceptibility test

*Staphylococcus* isolates were analyzed for antimicrobial resistance using the broth microdilution method according to the Clinical and Laboratory Standards Institute guidelines (Clinical and Laboratory Standards Institute, 2018) for the following antibiotic agents obtained from Oxoid (Wesel, Germany): ampicillin, erythromycin, tetracycline, ciprofloxacin, chloramphenicol, gentamicin, cefuroxime, nitrofurantoin, cefquinome, and enrofloxacin. *Staphylococcus epidermidis* ATCC 35984 and *S*. *aureus* ATCC 29213 were used as control strains.

### 2.3. Identification of antibiotic-resistant isolates

Twenty-six antibiotic-resistant isolates (Table 1) were genetically identified by 16S rDNA sequence analysis. Total genomic DNA was extracted from pure culture using the optimized protocol of the commercial Roche High Pure PCR Template Preparation kit (Basel, Switzerland). The 16S rDNA coding region sequence was selected and amplified by PCR. The 20 μL reaction mixture included template DNA, forward primer, reverse primer, water, and Roche AptaTaq Fast PCR Master. 16S-F (5′-AGAGTTTGATCCTGGCTCAG-3′) and 16S-R (5′-ATGGTACCGTGTGACGGGCGGTGTGTA-3′) universal primers were used to amplify the 16S-rDNA gene. Sequence analysis was performed by Macrogen (Amsterdam, Netherlands) (Lange et al., 2015).

### 2.4. Biocide tolerance and efflux pump inhibition

Minimum inhibitory concentrations (MICs) were determined by the broth microdilution method in 96-well microtiter plates according to the recommendations from the Clinical and Laboratory Standards Institute (2018). The biocides used for these assays—sodium hypochlorite (active chlorine 10–15%), povidone-iodine, hexachlorophene [2,2′-methylenebis(3,4,6-trichlorophenol)], chlorhexidine, benzalkonium chloride, and triclosan—were obtained from Sigma-Alrich (Madrid, Spain). Briefly, serial 2-fold dilutions of each substance were incubated with bacterial suspensions adjusted to 5 × 10^5^ CFU/mL in Mueller–Hinton broth (Merck, catalog number: 110293). Microtiter plates were incubated at 37 °C, and visual readings were taken after 24–48 h of incubation. The effect of the efflux pump inhibitor reserpine (Sigma-Aldrich, Madrid, Spain) on antibiotic and biocide MIC values was evaluated by adding reserpine to the medium at a concentration of 25 μg/mL (Fernandes-Fuentes et al., 2014). A ≥4-fold decrease in MIC in the presence of reserpine suggested that the resistance could be attributed to a reserpine-sensitive efflux pump (Gibbons and Udo, 2000).

### 2.5. Real-time PCR detection of antibiotic resistance and efflux pump genes

The presence of efflux pumps and antibiotic resistance genes was determined by real-time PCR amplification using the LightCycler system (Roche). Antibiotic resistance primers for *tetK* and *tetL* for tetracycline, *bla* and *mecA* for β-lactam, *cat* for chloramphenicol resistance, and efflux pump primers *qacA/B*, *acrB*, *sugE*, *smr*, *efrA*, *efrB*, *mdeA*, *mepA*, *norA*, and *sepA* were designed using information obtained from the NCBI PubMed database^2^ for this study. *norE* and *norB* primer sequences were taken from Swick et al. (2011) and Patel et al. (2010), respectively (Table 2). The Roche FastStart Essential DNA Green Master kit was used for the experiment. The study used primer pairs for the targeted gene region and SYBR Green dye on the LightCycler 96 (Roche Diagnostics, Mannheim, Germany).

## 3. Results

From 132 food samples, 77 *Staphylococcus* spp. isolates were recovered (Table 1). Antibiotic resistance to one or more antibiotic was found in 26 isolates by the MIC method (Table 3). Isolates were sensitive to the erythromycin, ciprofloxacin, gentamicin, cefquinome, and enrofloxacin antibiotics. Of the 26 resistant isolates, 18 (69.2%) were resistant to tetracycline, 10 (38.5%) to cefuroxime, three (11.5%) to chloramphenicol, two (7.7%) to nitrofurantoin, and one (3.9%) to ampicillin (Table 3). These isolates were selected for genotypic identification of antibiotic resistance and efflux pump genes (Table 3). For biocide tolerance, 12 different concentrations were tested for all biocides. Unlike other biocides, sodium hypochlorite and povidone-iodine concentrations were prepared using the doses used in commercial products (Table 4). MIC ranges of six biocides are given in Table 4.

The efflux pump inhibitor reserpine reduced the MIC value between 4- and 256-fold in all cefuroxime-resistant bacteria (Table 3). When reserpine was added, the MIC value decreased 4-fold or more in 5 (27.8%) of the tetracycline-resistant isolates and in both nitrofurantoin-resistant isolates, while there was no change in the MIC value in chloramphenicol- and ampicillin-resistant isolates. The effect of reserpine on biocide MIC values is shown in Table 4. Reserpine reduced biocide tolerance to triclosan (46.7%) and povidone-iodine (32%) most effectively.

Antibiotic resistance and efflux pump genes determined by real-time PCR are given in Table 3. Antibiotic resistance genes *tetK* were found in 17 isolates, *bla* in 5 isolates, and *mecA* in 1 isolate, while *tetL* and *cat* genes were not found in any of the tested isolates. The efflux pump genes identified with high frequency were *norE* (80.8%), *smr* (88.5%), *efrA* (84.6%), *efrB* (80.8%), *mdeA* (84.6%), *sepA* (53.8%), and *sugE* (50%). The *qacA/B* gene was not detected in any of the tested isolates.

## 4. Discussion

Antibiotic and biocidal agents used for hygiene, treatment, and prophylaxis in the veterinary and food industries exert selective pressure, leading to the development and spread of antimicrobial-resistant bacteria in animals and humans (Endale et al., 2023). Many studies have shown that biocides and antibiotics have similar mechanisms of action on bacteria, and that the resistance mechanisms that develop in bacteria may also be the same. Therefore, biocides can promote the spread of antibiotic-resistant bacteria, and exposure to biocides at subinhibitory concentrations can lead to cross resistance with antibiotics (Sousa et al., 2025).

In this study, resistance to at least one antibiotic was identified in 26 of the 77 *Staphylococcus* isolates. Since the study aimed to investigate efflux pump proteins, a common antibiotic and biocide resistance mechanism in foodborne *Staphylococcus* spp., further analysis was performed on these 26 antibiotic-resistant isolates. The antibiotics with the most resistance were tetracycline (69.2%) and cefuroxime (38.5%), consistent with the literature (Ghabbour et al., 2022; Taddese et al., 2025). Of the 18 tetracycline-resistant isolates, 17 had the *tetK* gene, which encodes a type of efflux pump protein known to be associated with tetracycline resistance and from the MFS family (Roberts., 2005). None of the isolates had the *tetL* gene. Reserpine reduced tetracycline resistance by 4-fold or more in 5 (27.8%) of the tetracycline-resistant isolates. This suggests that tetracycline resistance in *Staphylococcus* spp. isolated in our study may be due to the presence of the *tetK* gene, although a small portion of this resistance is due to reserpine-sensitive efflux proteins.

The *bla* gene causes resistance in *Staphylococcus* spp. by producing the beta-lactamase enzyme and enzymatically breaking down antibiotics such as penicillin and cephalosporin. The *mecA* gene, on the other hand, causes antibiotic resistance by inhibiting the entry of antibiotics into the cell by enabling the production of alternative proteins to penicillin-binding proteins (PBPs), which are the receptors for beta-lactam antibiotics (Tahmasebi et al., 2017). In our study, the *bla* gene was positive in 5 (50%) of the isolates identified as resistant to cefuroxime, while the *mecA* gene was detected in only one isolate showing resistance to both cefuroxime and ampicillin. The *mecA*-positive isolate was negative for the *bla* gene. This suggests that the beta-lactam antibiotic resistance of this isolate may have developed due to a change in the PBP protein caused by the *mecA* gene, not in beta-lactamase production. Ghabbour et al. (2022) examined the prevalence of the *mecA* and *blaZ* genes in *S*. *aureus* strains isolated from retail foods in Egypt. The results showed a lower prevalence of *mecA* (34%) and a higher prevalence of *blaZ* (82%). They suggested that some phenotypic beta-lactam resistance may be associated with *mecA*, even in the absence of *blaZ* (Ghabbour et al., 2022). The results of this study are similar to and support our findings.

The efflux pump inhibitor reserpine reduced the MIC values of all cefuroxime-resistant bacteria 4- to 256-fold, providing strong evidence that cefuroxime resistance in *Staphylococcus* spp. isolated from animal food sources originated from the efflux pump system. Similarly, Costa et al. (2013) reported that administration of reserpine to multidrug-resistant *S*. *aureus* isolates resulted in 2- to 128-fold reductions in antibiotic MIC values, supporting the role of efflux pump activity in beta-lactam resistance. Similar studies support the hypothesis that cefuroxime resistance in our study is mainly due to efflux pump activity (Huet et al., 2008; Costa et al., 2013). The *cat* resistance gene, which encodes the chloramphenicol acetyltransferase enzyme (Schwarz et al., 2004), was not detected in any of the 3 chloramphenicol-resistant *Staphylococcus* spp. isolates in our study. In addition, the chloramphenicol MIC value in these isolates was not affected by reserpine. This indicates that the resistance may not be due to the reserpine-sensitive efflux pump.

Hughes and Ferguson (2017) used 198 clinical *S*. *aureus* isolates and the agar dilution method to show the MIC values for chlorhexidine were between 0.5 and 8 mg/L. The MIC values for triclosan were between <0.06 and 16 mg/L. Compared to our study (Table 4), the *Staphylococcus* isolates had slightly higher MIC values for chlorhexidine. However, our MIC values for triclosan were much higher: 512–64 μg/mL in seven isolates and 32–4 μg/mL in two isolates. These results suggest that biocidal use in the food and livestock sectors is somewhat uncontrolled.

Marquez et al. (2017) screened 80 gram-positive bacteria isolated from various cheese samples produced by small and medium-sized enterprises in Spain, including *Bacillus*, *Enterococcus*, and lactic acid bacteria. They found that benzalkonium chloride MIC values were 2.5–50 mg/L, triclosan MIC values were 2.5–7.5 mg/L, and hexachlorophene MIC values were 5–10 mg/L. Although the MIC values for benzalkonium chloride were similar to those in our study, the MIC values for triclosan and hexachlorophene were much higher in our study. Marquez et al. (2017) noted that these values indicate reduced biocide sensitivity. Therefore, the isolates in our study also have significantly reduced biocide sensitivity.

Jomha et al. (2014) examined biocidal resistance of 86 gram-positive bacteria isolated from a hospital in Lebanon. Serial dilutions based on commercially available biocide concentrations were performed. Of the 31 *S*. *aureus* isolates, 12.9% were resistant to quaternary ammonium compound-containing disinfectants, 20.1% were resistant to sodium hypochlorite, and none were resistant to povidone-iodine. Of the 31 coagulase-negative *Staphylococcus*, 6.5% were resistant to quaternary ammonium compound-containing disinfectants and sodium hypochlorite; no resistance to povidone-iodine was observed.

Fernandes-Fuentes et al. (2014) collected 11 *Bacillus*, 25 *Enterococcus*, and 10 *Staphylococcus* isolates from organic foods. The MIC values of various biocidal substances decreased 4-fold or more in four isolates for benzalkonium chloride, 14 isolates for hexachlorophene, 19 isolates for chlorhexidine, and two isolates for triclosan when 25 μg/mL of reserpine was added to the medium. In our study, reserpine most effectively reduced resistance to povidone-iodine and triclosan (Table 4). This indicates that resistance to other biocidal substances, whose MIC values decrease with the addition of reserpine, particularly povidone-iodine and triclosan, may have developed with the reserpine-sensitive efflux pump system.

*norA*, *mdeA*, *mepA*, *sepA*, *smr*, and *qacA*/*B* genes have been studied in clinical isolates of *S*. *aureus*, especially methicillin-resistant/susceptible *S*. *aureus* (MRSA/MSSA). The protein products of these genes are efflux pumps, which are often involved in the development of resistance to antimicrobial agents (Hassanzadeh et al., 2020). Other genes *acrB*, *efrA*, and *efrB* have also been identified in gram-negative bacteria (e.g., *Escherichia coli* and *Klebsiella*) and contribute to the removal of antimicrobials from the cell (Fuentes et al., 2014).

Hassanzadeh et al. (2017) found the *mecA* gene in all 60 clinical MRSA isolates they collected in Tehran. They also found the *mdeA* gene in 43 isolates, the *mepA* gene in 37, the *norA* gene in 22, the *norB* gene in one, the *sepA* gene in 19, and the *smr* gene in 11 (Hassanzadeh et al., 2017). The results for genes other than *mecA* are similar to those in our study (Table 3). Molecular analysis confirmed the presence of multiple efflux pump genes in the resistant isolates. The most prevalent were *smr*, *efrA*, *efrB*, *mdeA*, and *norE*; these were detected in over 80% of the tested strains. Similar distributions of these genes have been observed in hospital and foodborne *Staphylococcus* isolates worldwide (Costa et al., 2013; Hassanzadeh et al., 2020).

PCR/qRT-PCR analyses of *S*. *aureus* clinical isolates showed that the prevalence of chromosomal genes *norA*, *norB*, *norC*, *mepA*, *sepA*, and *mdeA* was over 90% (Suma et al., 2023). In comparison, plasmid-borne genes *qacA/B* (approximately 20%) and *smr* (8–30%) were less common. These gene clusters (e.g., *norA* + *norB* + *norC* + *mepA* + *sepA* + *mdeA*) have been associated with widespread microbial resistance in MRSA/MSSA strains characterized by ciprofloxacin, vancomycin, and *mecA*, leading to clinical treatment challenges (Suma et al., 2023). Our research results indicate that efflux pump genes are also frequently observed in animal-based foodborne bacteria, emphasizing that transferring these genes to human pathogens through gene transfer could lead to significant problems.

The presence of multiple efflux pump genes in isolates from food products could contribute to the spread of antimicrobial resistance throughout the food chain. However, limitations such as the lack of gene expression data and the relatively small sample size in our study should be considered. Future studies that include transcriptomic and efflux activity analyses are needed to further elucidate the contribution of these genes to resistance phenotypes.

## 5. Conclusion

This study showed the high prevalence of efflux pump-related antimicrobial resistance genes in *Staphylococcus* spp. isolated from milk and meat samples in Ankara, Türkiye. The detection of *smr*, *efrA*, *efrB*, *mdeA*, and *norE* genes in a majority of resistant isolates, along with the significant reduction in MIC values by reserpine, indicates that efflux-mediated mechanisms play a key role in both antibiotic and biocide resistance. The absence of the *qacA/B* gene, despite widespread resistance, suggests possible regional or ecological differences in gene dissemination. The frequent occurrence of the *tetK* and *bla* genes, and the identification of the *mecA* gene in one isolate, further reflect the complexity of resistance determinants in foodborne *Staphylococcus* spp.

The decreased susceptibility to commonly used biocides, especially triclosan and povidone-iodine, provides information about the excessive and unnecessary use of these substances in food-related environments and raises the issue that this situation may contribute to the selection of resistant strains. The findings of the study highlight the need for better regulation and more appropriate use of antimicrobial agents in the veterinary and food sectors.

Although this study provides significant insights into the presence of resistance genes, further research, including gene expression analysis, transcriptomic profiling, and efflux pump functional assays, is essential to clarify the contribution of these genes to phenotypic resistance. Surveillance of efflux pump gene prevalence in foodborne bacteria is critical for anticipating public health risks and guiding evidence-based antimicrobial stewardship strategies.

## Figures and Tables

**Table 1:** Distributions of *Staphylococcus* spp. isolated from dairy and meat samples.

Food Sample	Number of samples	*Staphylococcus* spp.	
		Number of isolates	%
Cheese	60	51	66.2
Raw milk	13	16	20.8
Chicken	18	5	6.5
Calf meat	41	5	6.5
Total	132	77	100

**Table 2:** Primer sequences of efflux pump genes used for real-time PCR.

Primer (Source of sequence)	Sequence	
norB (Swick et al.2011)	F	GTAATGGTACTAATTATGATTCGTGTTGG
	R	CTGGCAAGAAAGTTAATGAAATGAGAC
norE (Patel et al.2010)	F	CTGGCGGCAGCGGTAA
	R	TGCCATACAGACACCCACCATA
acrB (This study)	F	ACCAAGTTCATCCACGCCTA
	R	AAAGGCTGGGCAAATGACTG
sugE (This study)	F	ACACAGTGATTGGCATGATTG
	R	GCATCCCCACAAAGAACCAA
efrA (This study)	F	GGAGCGGGACTGTTCAAATC
	R	GAATCCGTGAAGCCGAAACC
efrB (This study)	F	TCCTAGATGAGCCAGAAGAAGA
	R	GTCCCACAATCGCAACCATT
qacA/B (This study)	F	GCTGCTGTTGAAGAGTCTATGT
	R	TGCAAGCTGTTTTATCCCCG
smr (This study)	F	AGATCAGGCACCTTCAACGA
	R	GCATGGCGAAAATCCGTAGA
mdeA (This study)	F	TCCATACAATAACCCGCCGA
	R	TCGCTTTTCCATGTTGTCGC
mepA (This study)	F	TTTAGGGGCGAGAGGTGAAA
	R	TGATTGCAGTACCCAAAGCTG
norA (This study)	F	TGGGGCACTTTTCCAAATCT
	R	AGTTCAATCCGCCTGCAAAG
sepA (This study)	F	TCATGATACCTCTGACGGCA
	R	ATAAGTACGCCTGCACCCAT
bla (This study)	F	AGCAGATAAGAGTGGACAAGC
	R	GCTGTTTCGCTGACTACTTTG
mecA (This study)	F	GGCAGACAAATTGGGTGGTT
	R	TGAAGCAACCATCGTTACGG
tetK (This study)	F	TGTCTTGATTGCCCACGTTC
	R	TGCAACAGTTCACATGGACG
tetL (This study)	F	AGGAAGCAGAGTTCAGCCAT
	R	TAAGGGCACAAATCGCATCG
cat (This study)	F	TAACCTGCCCCGTTAGTTGA
	R	TTGTGCGTTGAGAAGAACCC

**Table 3:** Antibiotic resistance phenotype, antibiotic resistance, and efflux pump gene profile of *Staphylococcus* strains from dairy and meat samples.

	Origin of samples	Code of isolates	Species	Antibiotic resistance efflux pump gene profile
1	Cheese	P49-2	*S.saprophyticus*	CXM(4)* bla,norE,sugE,smr,efrA,efrB,mdeA
2	Cheese	P24-1	*S.saprophyticus*	CXM(256),TE tetK,norE,smr,efrA,efrB,mdeA,norB,sepA
3	Cheese	P2-2	*S.saprophyticus*	CXM(256) norE,smr,efrA,efrB,mdeA
4	Cheese	P23-1	*S.aureus*	CXM(64),TE(8) tetK,acrB,norE,efrB,mdeA
5	Cheese	P20-1	*S.saprophyticus*	CXM(64) acrB,smr,efrA,mdeA
6	Raw milk	S3-1	*S.aureus*	AM,CXM(256) mecA,mdeA,norB
7	Raw milk	ES-14	*S. condimenti*	CXM(512) bla,norE,sugE,smr,efrA,efrB,mdeA,sepA
8	Minced meat	K11-1	*S.xylosus*	CXM(64),TE tetK,bla,acrB,norE,smr,efrA,efrB,mdeA,sepA
9	Raw milk	ES-18	*S.succinus*	TE(4),C tetK,norE,sugE,smr,efrA,efrB
10	Raw milk	ES-20	*S.saprophyticus*	TE tetK,acrB,norE,smr,efrA,efrB
11	Cheese	P49-3	*S.saprophyticus*	CXM(128),TE tetK,bla,norE,sugE,smr,efrA,efrB,mdeA,norA,norB,sepA
12	Raw milk	ES-15	*S.succinus*	TE tetK,acrB,norE,sugE,smr,efrA,efrB,mdeA,norB,sepA,mdeA
13	Chicken	T1-1	*S. pasteuri*	TE tetK,sugE,smr,efrA,mdeA
14	Raw milk	S4-2	*S.aureus*	TE acrB,sugE,smr,efrA,efrB,mdeA,mepA,norA,norB,sepA
15	Minced meat	K6-1	*S.saprophyticus*	TE(8) tetK,norE,sugE,smr,efrA,mdeA,norA,sepA
16	Raw milk	ES-11	*Staphylococcus succinus subsp. casei*	TE tetK,norE,smr,efrA,efrB
17	Raw milk	ES-8	*S.succinus*	TE,C tetK,norE,smr,efrB,mdeA,mepA
18	Minced meat	K5-1	*S.saprophyticus*	TE(4) tetK,smr,efrA,mdeA,norA,sepA
19	Raw milk	ES-9	*S.succinus*	TE(4) tetK,norE,sugE,smr,efrA,efrB,mdeA,norA,sepA
20	Chicken	T18-1	*S.aureus*	TE tetK,acrB,norE,smr,efrA,efrB
21	Raw milk	S6-1	*S.aureus*	TE tetK,acrB,norE,sugE,efrB,mdeA,mepA,norB,sepA
22	Raw milk	S6-11	*S.aureus*	TE tetK,norE,sugE,smr,efrA,efrB,mdeA,sepA
23	Cheese	P2-1	*S.saprophyticus*	TE tetK,norE,sugE,smr,efrA,efrB,mdeA,norA,norB
24	Cheese	P44-1	*S.xylosus*	CXM(128) bla,norE,smr,efrA,efrB,mdeA,sepA
25	Raw milk	S8-1	*S.saprophyticus*	F(4) norE,sugE,smr,efrA,efrB,mdeA,norA,sepA
26	Cheese	P46-1	*S.xylosus*	C, F(16) norE,smr,efrA,efrB,mdeA,sepA

AM: ampicillin, C: chloramphenicol, CXM: cefuroxime, F: nitrofurantoin, TE: tetracycline,

* numbers in parentheses indicate the MIC reductions (fold) compared to the MICs of the isolate grown in the absence of reserpine.

**Table 4:** MIC value ranges of biocides on *Staphylococcus* spp. isolates.

Biocide	MIC Ranges (n=26^a^)		
	512-64	32-4	2-0.25
Benzalkonium chloride (BC) (μg/mL)	1(1)^b^	3	10
Hexachlorophene (HC) (μg/mL)	1(1)	-	-
Chlorhexidine (CH) (μg/mL)	-	2(1)	24
Triclosan (TC) (μg/mL)	7(6)	2(1)	6

	**50-6.25**	**3.125-0.391**	**0.195-0.025**
Sodium Hypochlorite (SC) (mg/mL)	8 (3)	16	1
Povidone-iodine (PI) (g/L)	23(7)	2(1)	-

a Isolates with no MIC values were determined as biocide susceptible.

b Numbers in parentheses indicate the number of isolates with 4-fold or more reduction in MIC value obtained with reserpine.

## Footnotes

1 T.C. Resmî Gazete (2011). Turkish Food Codex Microbiological Criteria Regulation [online]. Website: https://www.resmigazete.gov.tr/eskiler/2011/12/20111229m3-6.htm [accessed 04 November 2019].

2 NCBI (2024). PubMed Database Overview [online]. Website: https://www.ncbi.nlm.nih.gov/pubmed/ [accessed 04 November 2019].
